# The dual role of skin-resident Tregs: balancing autoimmunity in vitiligo and immune evasion in melanoma

**DOI:** 10.3389/fimmu.2026.1935158

**Published:** 2026-10-28

**Authors:** Xi Chen, Can Chen, Jianqiang Yang

**Affiliations:** 1 First Affiliated Hospital of Huzhou University, Huzhou, China; 2 Huzhou Central Hospital, Fifth School of Clinical Medicine of Zhejiang Chinese Medical University, Huzhou, China; 3 Huzhou Central Hospital, Affiliated Central Hospital of Huzhou University, Huzhou, China

**Keywords:** autoimmunity, immune tolerance, immunotherapy, melanoma, regulatory T cells (Tregs), vitiligo

## Abstract

Skin-resident regulatory T cells (Tregs) are specialized tissue-resident lymphocytes essential for maintaining skin immune homeostasis and tolerance. However, their functional plasticity allows them to drive diametrically opposing clinical outcomes depending on the local microenvironment. This review comprehensively examines the dual and divergent roles of skin-resident Tregs in two immunologically distinct diseases: vitiligo and melanoma. In vitiligo, an autoimmune condition characterized by the destruction of epidermal melanocytes, quantitative Treg deficiencies, impaired skin homing via the CCL22/CCR4 axis, and phenotypic instability—shifting toward a dysfunctional, pro-inflammatory Th1-like state—precipitate the breakdown of self-tolerance. This permits unrestrained autoreactive CD8+ cytotoxic T cell activity. Conversely, in melanoma, the tumor microenvironment (TME) actively recruits and metabolically reprograms Tregs into a highly suppressive state. These intratumoral Tregs upregulate inhibitory receptors (e.g., TIM-3, PD-1, CTLA-4) and secrete potent immunosuppressive cytokines, facilitating immune evasion by exhausting anti-tumor effector responses. By dissecting the mechanistic parallels and critical differences in Treg trafficking, phenotypic plasticity, and metabolic adaptation, this review provides a unified framework for understanding context-dependent immune regulation. Furthermore, we highlight emerging disease-specific therapeutic strategies, emphasizing approaches to replenish and enhance Treg function to restore immune tolerance in vitiligo, while selectively depleting or functionally impairing intratumoral Tregs to unleash anti-tumor immunity in melanoma.

## 1 Introduction

Regulatory T cells (Tregs), a specialized lineage of CD4+ T cells pioneered by the 2025 Nobel Laureates, are defined by the expression of the transcription factor Forkhead box P3 (FOXP3) and are essential for maintaining immune homeostasis and preventing autoimmunity (1, 2). The critical importance of these cells is underscored by the severe, multi-organ autoimmune pathology observed in patients with immune dysregulation, polyendocrinopathy, enteropathy, X-linked (IPEX) syndrome, a rare X-linked disorder caused by loss-of-function mutations in *FOXP3* (3). Beyond their well-characterized role in suppressing autoreactive T cells in lymphoid organs, Tregs also populate peripheral non-lymphoid tissues, where they acquire unique phenotypes and perform tissue-specific functions that extend beyond classical immune suppression (4–6). These tissue-resident Tregs contribute to the maintenance of tissue homeostasis, promote repair, and regulate the local microenvironment through interactions with both immune and non-immune cells (3, 4).

The skin harbors a distinct population of tissue-resident Tregs—specifically termed skin-resident Tregs in this review—that are integral to cutaneous immune regulation and tissue integrity (5, 7). Skin-resident Tregs exhibit a transcriptional profile that distinguishes them from their circulating counterparts, enabling them to respond to local cues and perform functions such as modulating wound healing, regulating hair follicle cycling, and controlling inflammation (4, 8, 9). The dual capacity of Tregs to both suppress immune responses and support tissue repair positions them as central players in the pathogenesis of two clinically significant and immunologically opposing skin conditions: vitiligo and melanoma.

Vitiligo is an autoimmune skin disease characterized by the progressive destruction of epidermal melanocytes by autoreactive CD8+ cytotoxic T cells, resulting in disfiguring depigmented patches (10–12). The breakdown of self-tolerance in vitiligo is increasingly attributed to quantitative and qualitative deficiencies in Tregs (10, 12, 13). Patients with active vitiligo exhibit reduced Treg frequencies in peripheral blood, impaired suppressive function, decreased expression of FOXP3 and immunoregulatory cytokines such as IL-10 and TGF-β, and a skewed differentiation toward pro-inflammatory Th1-like phenotypes (10, 12–15). These defects collectively create an environment permissive for melanocyte-directed autoimmunity.

In melanoma, the most lethal form of skin cancer, Tregs are co-opted by the tumor to facilitate immune evasion and disease progression (16–18). Accumulation of Tregs within the tumor microenvironment (TME) is a well-documented phenomenon, and a high ratio of CD8+ T cells to Tregs within tumors is predictive of improved patient survival (16–19). Tregs in melanoma suppress anti-tumor immune responses through multiple mechanisms, including the production of immunosuppressive cytokines, expression of inhibitory receptors, and metabolic disruption of effector T cells (18, 20, 21). The presence of Tregs thus represents a major hurdle for effective immunotherapy, and strategies to deplete or functionally impair these cells are under active investigation (17, 18, 22).

This review synthesizes current knowledge on the biology of skin-resident Tregs and examines their divergent roles in vitiligo and melanoma. By exploring the mechanistic parallels and critical differences in Treg function across these two diseases, we aim to provide a comprehensive framework for understanding how the same cell type can drive opposing clinical outcomes. Furthermore, we discuss the therapeutic implications of Treg modulation, highlighting strategies to enhance Treg function in vitiligo while selectively inhibiting their suppressive activity in melanoma.

## 2 Skin-resident regulatory T cells: biology and tissue-specific functions

The concept of tissue-resident Tregs has fundamentally expanded the understanding of Treg biology beyond the traditional view of these cells as circulating suppressors confined to lymphoid organs (4, 5). Historically, early immunological paradigms from the late 1990s and early 2000s posited that Tregs primarily exerted their suppressive functions within secondary lymphoid organs. However, pioneering studies subsequently challenged this dogma, demonstrating that substantial numbers of T cells, including functional Tregs, reside long-term in healthy human skin (23). This historical paradigm shift laid the groundwork for modern single-cell technologies to uncover the precise molecular signatures of these cells. Tissue-resident Tregs are defined by their stable residence in non-lymphoid tissues, where they do not recirculate through blood or lymphatics and acquire a unique phenotype shaped by the local microenvironment (4, 5, 7). A core transcriptional program of tissue residency, shared across different tissues and species, has been identified. This ubiquitous tissue-Treg module is heavily reliant on transcription factors such as BATF and IRF4, which broadly orchestrate effector differentiation, survival, and interaction with local cell types (3, 4). However, significant differences emerge in Tregs destined for the skin, as they must adapt to the unique cutaneous metabolic and environmental cues. Molecularly, skin-resident Tregs are distinguished by the pronounced dependence on the transcription factor GATA3, which synergizes with IRF4 to maintain localized phenotypic stability and immune suppressive function (24, 25). Furthermore, unlike their counterparts in visceral adipose tissue or the gut mucosa, skin-destined Tregs exhibit a distinct transcriptomic signature characterized by the upregulation of cutaneous homing and retention molecules, most notably cutaneous lymphocyte-associated antigen (CLA), alongside specific chemokine receptors such as CCR4 and CCR8 (26).

In the skin, Tregs represent a prominent and quantitatively substantial component of the resident immune cell population. Specifically, studies evaluating normal human skin have established that FOXP3+ Tregs constitute approximately 20% of the entire resident CD4+ T cell pool in the steady state, a proportion significantly higher than that observed in peripheral blood (5, 7, 27). Notably, the distribution of these resident Tregs exhibits marked regional heterogeneity depending on the skin area. Because skin Tregs preferentially cluster around the bulge region of hair follicles to regulate stem cell cycling, their density is substantially higher in hair-bearing cutaneous sites (such as the scalp and trunk) compared to glabrous, non-hairy skin (such as the palms and soles) (28). Ontogenically, this cutaneous pool comprises a mix of both thymus-derived Tregs (tTregs), which typically maintain tolerance to self-antigens, and peripherally induced Tregs (pTregs), which are often generated in response to skin commensal microbiota and environmental exposures (29). Furthermore, skin-resident Tregs are highly stable and long-lived. Having acquired a memory-like phenotype, they can persist within the cutaneous microenvironment for extended periods to provide durable local immune regulation, complemented by a slow but continuous replenishment from the circulation (27, 30). Skin-resident Tregs are characterized by the expression of markers such as CD69, CD103, and CCR8, which facilitate their retention and function within the cutaneous microenvironment (4, 31). Single-cell transcriptomic analyses have revealed that skin Tregs exhibit a distinct gene expression profile compared to their counterparts in blood or lymphoid tissues, including the upregulation of genes associated with tissue repair, such as amphiregulin (*Areg*) and proenkephalin (*Penk*) (4, 32). These cells are strategically localized near hair follicles and within the epidermis and dermis, positioning them to interact with epithelial cells, fibroblasts, and other immune cells (4, 33, 34).

The functions of skin-resident Tregs extend well beyond immune suppression. Upon tissue injury, skin Tregs rapidly accumulate at the wound site and promote repair through the production of amphiregulin, an epidermal growth factor family member that acts on epithelial cells and fibroblasts to enhance proliferation and tissue regeneration (4, 9, 35). This repair-promoting function is mediated in part through the IL-33/ST2 axis, as IL-33 released from damaged epithelial cells activates ST2-expressing Tregs, leading to amphiregulin production and downstream tissue repair processes (4, 33, 35). Studies using Treg-specific deletion of *Jag1* (encoding Jagged-1), a Notch signaling ligand, have demonstrated that Jagged-1 expression on skin Tregs is required for efficient wound closure, further highlighting the complexity of Treg-mediated tissue repair mechanisms (9).

Skin Tregs also play a critical role in maintaining epithelial homeostasis and regulating stem cell function. Through interactions with epithelial stem cells, Tregs can modulate differentiation and regeneration programs (8). Jagged-1-expressing skin Tregs have been shown to promote hair follicle regeneration by signaling through the Notch pathway on hair follicle stem cells (9). In the context of carcinogenesis, the IL-33/ST2 axis and skin Tregs have been implicated in protecting against the development of cutaneous squamous cell carcinoma by regulating epithelial cell differentiation and minimizing mutational load after exposure to environmental carcinogens (33).

The recruitment and maintenance of Tregs in the skin are governed by specific chemokine-receptor interactions. The chemokine CCL22, which binds to CCR4 expressed on Tregs, is a key mediator of Treg homing to the skin (36–38). In vitiligo, reduced expression of CCL22 in the skin correlates with decreased Treg infiltration, suggesting that impaired chemokine signaling contributes to the local Treg deficiency observed in this disease (36, 37). In melanoma, elevated levels of CCL22 and CCL1 within the TME promote the recruitment of CCR4+ Tregs, contributing to the establishment of an immunosuppressive milieu (38). Additionally, CCR6 has been identified as another chemokine receptor important for Treg migration to the skin, as Tregs lacking CCR6 exhibit a reduced capacity to migrate to the skin and suppress depigmentation in vitiligo models (39). The chemokine receptor CCR5 has also been shown to facilitate proper positioning of Tregs within the skin, which is critical for their suppressive function *in vivo* (40).

The heterogeneity of skin Tregs is increasingly appreciated. Functional diversity among Treg subsets in the skin has been revealed through single-cell analyses, with subpopulations exhibiting differential expression of CD25, CD39, Granzyme B, cytotoxic T-lymphocyte-associated protein 4 (CTLA-4), and Neuropilin-1 (41). This heterogeneity is linked to trafficking behavior, with Tregs that remain in the skin displaying a distinct phenotype compared to those that are newly recruited or emigrating to draining lymph nodes (41). The identification of CD81 as a marker of highly activated skin Tregs that express both Penk and amphiregulin further underscores the complexity of the skin Treg compartment (32). Understanding this functional heterogeneity is crucial for developing targeted therapeutic strategies that modulate specific Treg subsets in disease contexts.

## 3 The role of Tregs in vitiligo: from dysfunction to autoimmune pathogenesis

Vitiligo is a prototypical autoimmune disease in which the breakdown of self-tolerance leads to the targeted destruction of melanocytes by autoreactive CD8+ T cells (10, 11, 42). The pathogenesis of vitiligo is multifactorial, involving genetic susceptibility, oxidative stress, and immune dysregulation (42, 43). Among the immune components, Tregs have emerged as central players in the failure of peripheral tolerance that permits autoimmunity to proceed (10–12, 44).

A substantial body of evidence, supported by multiple meta-analyses, indicates that vitiligo patients exhibit both quantitative and qualitative defects in Tregs (12, 13). A comprehensive meta-analysis of 30 studies involving over 1200 vitiligo patients and 1100 controls demonstrated a significant reduction in peripheral blood Treg frequency in patients compared to healthy controls (13). This reduction was particularly pronounced in patients with active disease, suggesting that Treg deficiency is linked to disease progression (13, 15). The suppressive capacity of Tregs is markedly impaired in vitiligo, with a specific defect in their ability to suppress CD8+ T cell proliferation and function (12, 13, 15). This functional impairment is associated with reduced expression of FOXP3 in both peripheral blood and lesional skin (12, 13, 15).

The molecular mechanisms underlying Treg dysfunction in vitiligo are complex and involve multiple pathways. Transcriptomic analyses have revealed that Tregs from vitiligo patients undergo a phenotypic shift toward a Th1-like profile, characterized by the expression of T-bet and IFN-γ (14, 45). These Th1-like Tregs retain FOXP3 expression but exhibit significantly reduced suppressive capacity. Furthermore, they are more readily chemoattracted by CXCL10 and CXCL16, which are elevated in the vitiligo microenvironment (14). The inflammatory milieu in vitiligo patients, particularly the presence of IFN-γ, appears to drive this conversion, as exposure of healthy Tregs to vitiligo patient serum can induce the Th1-like phenotype and impair their suppressive function (14). This suggests a vicious cycle in which the inflammatory environment further compromises Treg function, perpetuating autoimmune activity.

Defects in signaling pathways critical for Treg function have also been identified. The calcium-NFATc1 signaling pathway, which is essential for Treg activation and suppressive function, is disrupted in vitiligo Tregs (46–48). Reduced intracellular calcium levels, decreased expression of the calcium channel ORAI1, and diminished calcineurin and NFATc1 activity have been documented in Tregs from generalized vitiligo patients (47). These signaling defects are associated with reduced expression of critical downstream targets required for Treg-mediated suppression and survival. In the specific context of Treg function, granzyme B is utilized not for pathogen clearance, but to execute contact-dependent suppression by inducing apoptosis in autoreactive effector T cells and antigen-presenting cells (49). To prevent self-inflicted damage during this process, Tregs crucially rely on the co-expression of the granzyme B inhibitor SERPINB9, which protects them from autolysis and maintains their viability. Additionally, neuropilin-1 (NRP1) acts as a vital coreceptor that facilitates prolonged, stable interactions with dendritic cells and promotes Treg phenotypic stability in inflammatory milieus (50). The coordinated downregulation of these three molecules in vitiligo thus severely compromises both the suppressive capacity and longevity of Tregs. *In vitro* treatment of vitiligo Tregs with calcium can partially restore NFATc1 activity and improve suppressive capacity, suggesting a potential therapeutic avenue (47, 48). Similarly, treatment with the compounds harmine and kaempferol has been shown to enhance NFATc1 and FOXP3 expression and improve Treg suppressive function in vitiligo (46).

The enzyme heme oxygenase-1 (HO-1) has also been implicated in Treg dysfunction in vitiligo. HO-1 expression is reduced in Tregs from vitiligo patients, and its metabolite products are decreased in patient serum (51). Activation of HO-1 with hemin restores Treg function by upregulating IL-10 expression, indicating that HO-1 is a functional modulator of Treg activity in this disease (51).

The functional impairment of Tregs in vitiligo is further compounded by the dysregulation of key coinhibitory receptors, presenting a fascinating contextual divergence from melanoma. Under steady-state conditions, Tregs critically depend on inhibitory checkpoints, such as CTLA-4 and PD-1, to execute contact-dependent suppression. In vitiligo, the functional engagement of these networks is severely compromised. For instance, reduced expression or functional deficiency of CTLA-4—frequently linked to genetic polymorphisms—impairs the ability of Tregs to neutralize antigen-presenting cells. The role of PD-1 presents a particularly striking paradox. While PD-1 expression on Tregs in melanoma marks a hyper-suppressive state that drives tumor immune evasion, studies have demonstrated that the frequency of PD-1+ Tregs is paradoxically increased in the peripheral blood of patients with active generalized vitiligo, positively correlating with disease severity (52). In the context of vitiligo, this elevated PD-1 expression likely reflects a state of chronic overstimulation and subsequent cellular exhaustion, or a failed compensatory mechanism. Because the effective downstream PD-1/PD-L1 signaling axis is functionally defective in the vitiligo microenvironment, these “exhausted” PD-1+ Tregs are unable to halt autoreactive CD8+ T cells, rendering them susceptible to pro-inflammatory Th1-like conversion. Importantly, therapeutic interventions such as narrow-band UVB or emerging PD-L1 fusion proteins do not merely expand Treg numbers; they actively rescue these exhausted cells by re-engaging critical inhibitory networks, thereby reinforcing the barrier against autoreactivity (10, 53).

At the tissue level, the skin of vitiligo patients is characterized by a paucity of Tregs, particularly in lesional and perilesional areas (15, 36, 54). This local Treg deficiency is thought to be a critical factor that allows autoreactive CD8+ T cells to mediate melanocyte destruction. The reduced Treg infiltration into vitiligo skin has been attributed, at least in part, to decreased expression of the Treg-attracting chemokine CCL22 in the skin (36, 37). Circulating Tregs from vitiligo patients express normal levels of the CCL22 receptor CCR4, suggesting that the defect lies in chemokine production rather than Treg responsiveness (36). Supporting this concept, overexpression of *Ccl22* in the skin of vitiligo-prone mice promotes Treg infiltration and significantly reduces depigmentation (36, 37). The chemokine receptor CCR6 is also critical for Treg migration to the skin in vitiligo, as Tregs lacking CCR6 fail to suppress depigmentation despite normal systemic numbers (39).

The functional interaction between Tregs and other immune cells is also altered in vitiligo. Beyond their defective suppression of CD8+ T cells, Tregs from vitiligo patients exhibit impaired ability to suppress natural killer (NK) cell function (55). Mechanistically, this defect is primarily driven by impaired transforming growth factor-beta (TGF-β) signaling. Tregs in vitiligo display reduced production of soluble TGF-β and decreased expression of surface latency-associated peptide (LAP). As a result, these impaired Tregs fail to downregulate activating receptors, notably NKG2D, on NK cells and are unable to effectively inhibit NK-derived IFN-γ production. This breakdown in Treg-NK cell crosstalk unleashes NK cell cytotoxicity, contributing to increased NK cell activity and enhanced melanocyte destruction in generalized vitiligo (55). The imbalance between Tregs and effector cells extends to the Th17 axis, with elevated levels of IL-17 and IL-22 observed in vitiligo patients, further skewing the immune environment toward inflammation (12, 56).

The importance of Tregs in controlling vitiligo has been demonstrated in preclinical models. In the h3TA2 transgenic mouse model—a T cell receptor (TCR) transgenic model in which CD8+ T cells specifically recognize the tyrosinase epitope (Tyr368–376) presented by HLA-A*0201—mice develop spontaneous vitiligo driven by these tyrosinase-reactive T cells. In this model, adoptive transfer of purified Tregs or treatment with rapamycin can induce lasting remission, even in mice with established disease (56). Transfer of even small numbers of Tregs into depigmenting mice can effectively interfere with disease progression (36). These findings provide strong evidence that quantitative enhancement of Tregs can overcome autoimmune activity and restore tolerance.

## 4 The role of Tregs in melanoma: from immune tolerance to tumor progression

In stark contrast to vitiligo, where Treg deficiency promotes autoimmunity, melanoma is characterized by an overabundance of Tregs that actively suppress anti-tumor immunity and facilitate disease progression (16–18, 22). The accumulation of Tregs within the melanoma TME is a well-established phenomenon that correlates with poor prognosis and reduced survival (16, 17, 19, 57). The ratio of CD8+ T cells to Tregs within tumors has emerged as a critical prognostic indicator, with a higher ratio predicting better patient outcomes (16–18). This observation underscores the functional antagonism between effector T cells and Tregs within the tumor milieu.

The foundational concept of Treg-mediated tumor immune evasion was established by landmark studies in the early 2000s. Early foundational evidence revealed a profound enrichment of FOXP3-expressing CD4+CD25+ regulatory T cells within human metastatic melanoma lymph nodes and tumor sites, demonstrating their potent ex vivo capacity to suppress autologous anti-tumor effector cells (58). Building upon this historic foundation, contemporary research has elucidated that multiple mechanisms contribute to Treg accumulation in melanoma. The TME actively recruits Tregs through the secretion of chemokines such as CCL22 and CCL1, which bind to CCR4 expressed on Tregs (38, 59). Studies have documented a five-fold increase in CCL22 and CCL1 expression in melanoma-affected skin compared to unaffected skin, accompanied by robust infiltration of FOXP3+ T cells (38). Additionally, the proliferation and expansion of Tregs within the tumor are supported by local factors, including TGF-β and IL-10, which are abundant in the melanoma microenvironment (18, 60). Crucially, interleukin-2 (IL-2) plays a dual and fundamental role in this niche. Because Tregs constitutively express high levels of the high-affinity IL-2 receptor (CD25) but lack the ability to produce IL-2 themselves, they act as a metabolic “IL-2 sink”. Tregs aggressively consume the limited local IL-2 synthesized by activated effector cells. This metabolic consumption fuels their own robust expansion and functional stability. Concurrently, it starves local anti-tumor CD8+ T cells of this vital survival factor, executing a potent form of suppression via cytokine deprivation (61). The presence of CD8+ T cells themselves can drive Treg recruitment through the production of CCR4-binding chemokines and the induction of Treg proliferation, creating a feedback loop that amplifies immunosuppression (59).

Tregs in melanoma employ a diverse arsenal of suppressive mechanisms to inhibit anti-tumor immune responses. They produce immunosuppressive cytokines such as IL-10, TGF-β, and IL-35, which directly inhibit effector T cell function and promote a tolerogenic environment (60, 62). IL-35, in particular, has been shown to induce a potent regulatory population called iTR35 cells, which mediate suppression in an IL-35-dependent manner and contribute to Treg-mediated tumor progression (62). Tregs also express inhibitory receptors, including CTLA-4, PD-1, TIGIT, and TIM-3, which engage their ligands on antigen-presenting cells or effector T cells to deliver inhibitory signals (63–66). The expression of TIM-3 on intratumoral Tregs marks a highly suppressive subset that co-expresses PD-1 and is enriched in tumor tissues, contributing to T cell dysfunction (64). The transcription factor BATF has been identified as a key regulator of Treg activation in the TME, epigenetically controlling the expression of genes required for Treg suppressive function and stability (67).

The metabolic interplay within the TME profoundly influences Treg function. The hypoxic conditions that prevail in many solid tumors drive metabolic reprogramming of Tregs, promoting their survival, proliferation, and immunosuppressive activity (21). Tregs are particularly adept at utilizing alternative metabolic substrates to meet their energy demands in this challenging environment (21). The enzyme glutathione peroxidase 4 (Gpx4) is crucial for protecting activated Tregs from lipid peroxidation and ferroptosis, a form of regulated cell death (68). Treg-specific deletion of *Gpx4* leads to ferroptosis upon activation and enhances anti-tumor immunity, suggesting that targeting this pathway could selectively impair Tregs in the TME (68). AMP-activated protein kinase (AMPK), a key energy sensor, is required for Treg functional adaptation to metabolic stress in tumors, linking metabolic fitness to suppressive capacity (69).

The antigen specificity of tumor-infiltrating Tregs has been a subject of considerable interest. Studies have shown that Tregs within melanoma metastases display a diverse yet distinct TCR repertoire that overlaps with circulating Tregs but is distinct from conventional T cells (70). TCRs isolated from intratumoral Tregs exhibit specific reactivity against autologous tumors and mutated neoantigens, indicating that these Tregs are activated and clonally expanded in response to tumor antigens (70). This finding suggests that tumor-specific Tregs act in an antigen-selective manner, directly suppressing anti-tumor immune responses at the site of antigen encounter.

The presence of tumor antigen-specific Tregs in the peripheral blood of melanoma patients has also been documented (71). These circulating Tregs recognize a broad range of tumor antigens, including melanocyte differentiation antigens such as gp100 and TRP1, as well as cancer-testis antigens like NY-ESO-1 (71). They produce IL-10, express high levels of FOXP3, and suppress autologous CD4+ T cell responses in a contact-dependent manner (71). The existence of these systemic tumor-specific Tregs implies that Treg-mediated suppression is not confined to the TME but extends to peripheral immune compartments.

The prognostic significance of Tregs in melanoma has been extensively investigated. Elevated levels of circulating Tregs at the time of diagnosis are correlated with poor overall survival and disease-free survival in melanoma patients (57, 72). In uveal melanoma, intratumoral FOXP3+ Tregs are an independent prognostic marker for worse survival, particularly in cyclooxygenase-2 (COX-2)-positive tumors, suggesting a link between COX-2 expression and Treg induction (73). A Treg-related gene signature (TRGS) has been developed that can predict survival outcomes in melanoma patients, with high-risk scores associated with worse prognosis and immunosuppressive TMEs (74, 75). These signatures highlight the potential utility of Treg-related biomarkers for patient stratification and therapeutic decision-making.

The role of Tregs in modulating response to immunotherapy is a rapidly evolving area of research. Anti-CTLA-4 therapy, while initially thought to deplete Tregs in humans, has been shown to primarily increase intratumoral CD4+ and CD8+ T cell infiltration without significantly depleting FOXP3+ cells in the TME (76). In contrast, murine studies have demonstrated that the efficacy of anti-CTLA-4 antibodies depends on Fcγ receptor-mediated depletion of intratumoral Tregs by tumor-associated macrophages, highlighting species-specific differences in mechanism of action (77). Anti-PD-1 therapy leads to a decline in circulating PD-1+ Tregs, and patients who exhibit this decline have improved clinical outcomes, including better progression-free survival and reduced melanoma-specific death (78). However, PD-1 blockade can also paradoxically increase Treg proliferation and induce phosphorylated STAT3 (pSTAT3) expression, which is associated with reduced Treg suppressive function in patients who benefit from therapy (79).

The most striking clinical manifestation of the mechanistic intersection between these two diseases is the phenomenon of immune checkpoint inhibitor (ICI)-induced vitiligo. Clinically, the development of vitiligo-like depigmentation in melanoma patients undergoing ICI therapy (such as anti-PD-1 or anti-CTLA-4) is a well-documented immune-related adverse event that carries profound prognostic significance. Comprehensive clinical studies and meta-analyses have consistently demonstrated that the onset of ICI-induced vitiligo serves as a strong visible biomarker of robust systemic anti-tumor immunity, correlating with significantly prolonged progression-free and overall survival (80, 81). Mechanistically, this phenomenon elegantly illustrates the shared antigenic landscape and the delicate balance of Treg-mediated tolerance. ICIs not only reinvigorate exhausted effector T cells but also actively disrupt Treg-mediated peripheral tolerance. This therapeutic uncoupling of suppression unleashes cross-reactive CD8+ T cells that target shared melanocyte differentiation antigens (e.g., Melan-A, gp100) present on both malignant melanoma cells and healthy cutaneous melanocytes. Consequently, the implication for Tregs is profound: successfully crippling Treg suppressive function to achieve melanoma eradication inherently lowers the threshold for cutaneous self-tolerance, leading to the targeted autoimmune destruction seen in vitiligo. This clinical paradigm serves as the ultimate *in vivo* proof-of-concept for the “two sides of the same coin” hypothesis central to these two diseases.

## 5 Mechanistic parallels and divergences: how the same cell type shapes opposite outcomes

The contrasting roles of Tregs in vitiligo and melanoma provide a compelling illustration of context-dependent immune regulation. In both diseases, Tregs are central mediators of immune tolerance, yet the direction of their effect is diametrically opposite: in vitiligo, Treg deficiency permits autoimmunity, while in melanoma, Treg excess suppresses protective anti-tumor immunity (10, 11, 16, 17). Understanding the mechanistic parallels and divergences between these two scenarios offers valuable insights into the fundamental principles of Treg biology and may inform the development of disease-specific therapeutic strategies. A critical prerequisite for understanding these diametrically opposing outcomes is recognizing the dynamic phenotypic and functional shifts Tregs undergo across different compartments. Circulating Tregs, originating primarily from the thymus, typically express naive or central memory markers (e.g., CD62L, CCR7) and function to maintain baseline systemic tolerance (4, 30). Upon migrating into normal skin, these cells differentiate into true tissue-resident Tregs, acquiring a distinct transcriptomic signature (e.g., CLA, CCR4, GATA3, CD69, CD103) that facilitates not only localized immune tolerance but also tissue repair (e.g., amphiregulin production) and stem cell niche maintenance (4, 24, 26). However, in pathological states, this baseline tissue-resident profile is profoundly hijacked. In vitiligo, the skin-resident Treg pool is not only quantitatively depleted but qualitatively destabilized, shedding typical regulatory markers and acquiring a Th1-like profile (IFN-γ+, T-bet+) that fails to suppress local autoreactivity (14, 45). Conversely, in melanoma, the TME corrupts this trajectory; tumor-infiltrating Tregs (TITRs) are actively recruited and hyper-activated, expressing an extreme array of coinhibitory receptors (TIM-3, PD-1, TIGIT, high CTLA-4) and metabolic adaptations (e.g., GPX4 dependency, IL-2 deprivation) that far exceed the suppressive capacity of normal skin-resident Tregs (61, 64, 68). These critical multidimensional differences in origin, markers, and function are systematically synthesized in **Table 1**.

**Table 1:** Comparative analysis of treg populations: circulating, skin-resident, and disease-contextualized alterations.

Feature	Circulating tregs (systemic)	Healthy skin-resident tregs	Disease context alterations (vitiligo vs. melanoma)
Origin/Source	Primarily Thymus-derived (tTregs); some peripherally induced (pTregs)	Mixed origin (tTregs and commensal-induced pTregs); long-lived local persistence	Vitiligo: Impaired local recruitment due to ↓CCL22. Melanoma: Active recruitment (↑CCL22/CCL1) and robust local intratumoral expansion
Key Phenotypic Markers	CD4+, CD25+, FOXP3+, CD62L+, CCR7+	FOXP3+, GATA3+, CLA+, CCR4+, CCR8+, CD69+, CD103+	Vitiligo: Th1-skewed (T-bet+, IFN-γ+); ↓FOXP3 stability Melanoma: Hyper-suppressive subset (TIM-3+, TIGIT+, PD-1+, high CTLA-4+)
Primary Core Function	Maintenance of systemic immune tolerance; prevention of generalized autoimmunity	Local immune homeostasis; suppression of skin inflammation; promotion of wound healing (Areg) and hair follicle cycling	Vitiligo: Functional incompetence; failure to suppress cytotoxic CD8+ T and NK cells Melanoma: Extreme immunosuppression; acts as metabolic “IL-2 sink”; potent cytokine secretion (IL-10, IL-35, TGF-β)
Metabolic & Signaling State	Baseline metabolic and survival requirements in blood	Adapted to the cutaneous microenvironment and tissue repair pathways	Vitiligo: Disrupted calcium-NFATc1 signaling and ↓HO-1; driven by inflammatory stress rather than nutrient deprivation Melanoma: Highly adapted to hypoxia/ferroptosis stress (GPX4 dependence, AMPK signaling)

One of the most striking parallels is the importance of Treg localization and migration. In both vitiligo and melanoma, the chemokine-chemokine receptor axis involving CCL22 and CCR4 plays a critical role in directing Treg trafficking to the skin (36–38). However, the outcome of this recruitment differs dramatically due to the opposing goals of the immune response in each disease. In vitiligo, the paucity of CCL22 in the skin leads to insufficient Treg homing, creating a permissive environment for melanocyte-specific autoimmunity (36, 37). In melanoma, tumor-derived CCL22 actively recruits Tregs, establishing an immunosuppressive niche that protects the tumor from immune destruction (38). This divergence highlights how the same molecular pathway can be exploited for opposite effects depending on the disease context.

The functional state of Tregs also differs between the two diseases. In vitiligo, Tregs are characterized by instability and a propensity to convert to pro-inflammatory Th1-like phenotypes that express IFN-γ and lose suppressive capacity (14, 45). This functional impairment is driven by the Th1-skewed inflammatory environment, which is rich in IFN-γ and CXCL10 (14). In melanoma, while Tregs can also exhibit phenotypic plasticity, the dominant phenotype is one of enhanced suppressive function, with high expression of inhibitory receptors such as TIM-3, PD-1, and TIGIT, as well as potent immunosuppressive cytokine production (63–65). The TME actively promotes this highly suppressive state through factors such as TGF-β, IL-10, and metabolic cues (21, 60) (**Figure 1**).

**Figure 1:**
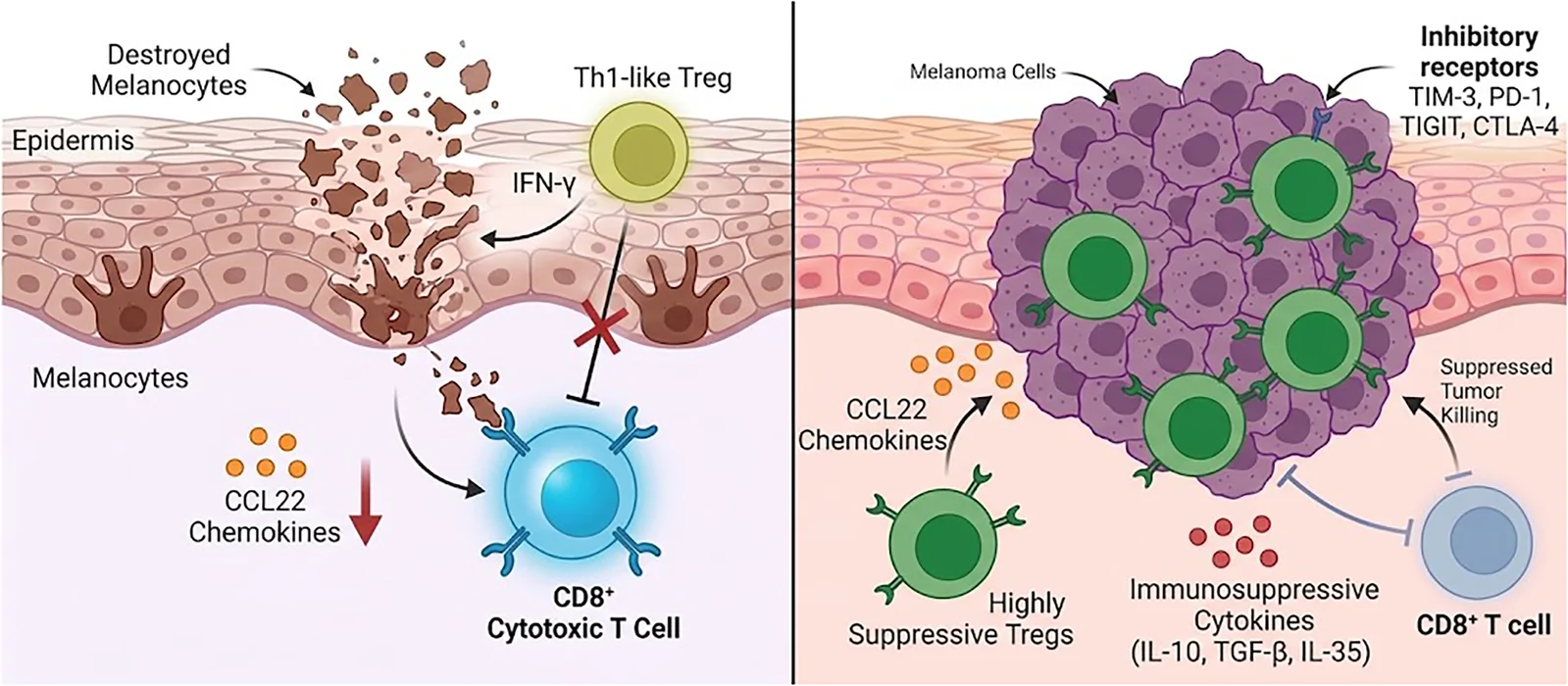
Mechanistic divergences of skin-resident regulatory T cells (Tregs) in the pathogenesis of vitiligo and melanoma. (Left) In vitiligo, a reduction in the chemokine CCL22 impairs the recruitment of Tregs to the skin. Within the local inflammatory milieu, Tregs undergo a phenotypic shift toward a dysfunctional Th1-like state, characterized by the production of IFN-γ and a marked loss of suppressive capacity. Consequently, these impaired Tregs fail to inhibit autoreactive CD8+ cytotoxic T cells, permitting the unhindered destruction of epidermal melanocytes. (Right) In melanoma, the TME actively recruits Tregs through the elevated secretion of CCL22 chemokines. Once localized within the tumor, these highly suppressive Tregs express an array of inhibitory receptors (including TIM-3, PD-1, TIGIT, and CTLA-4) and secrete potent immunosuppressive cytokines (such as IL-10, TGF-β, and IL-35). This robust regulatory network directly inhibits CD8+ T cell effector functions, thereby suppressing anti-tumor immunity and facilitating tumor immune evasion.

The metabolic demands placed on Tregs also differ between the two diseases. In the metabolically stressed TME, Tregs must adapt to hypoxia and nutrient deprivation to maintain their suppressive function, relying on pathways such as AMPK signaling and protection from ferroptosis (21, 68, 69). In vitiligo, while oxidative stress is a feature of the disease, the metabolic challenges are distinct, and Treg dysfunction is more related to signaling defects and phenotypic instability rather than an inability to survive in a harsh metabolic environment (43, 47, 51).

A comparative single-cell transcriptomic analysis of vitiligo and melanoma has provided direct insights into the divergent immune landscapes of these two diseases (82). In vitiligo, the immune microenvironment is dominated by CD8+ T cells, with IFN-γ-CXCL9/10-CXCR3 axis-mediated melanocyte elimination as a central feature. Stressed fibroblasts and keratinocytes amplify these pro-inflammatory signals. In melanoma, the microenvironment is regulated by Tregs and cancer-associated fibroblasts, leading to impaired cytotoxic function of CD8+ T cells (82). This analysis underscores how the balance between effector and regulatory cells dictates the outcome of the immune response in each disease.

The role of specific Treg subsets also differs. In vitiligo, the loss of tissue-resident Tregs and the emergence of dysfunctional Th1-like Tregs are key pathogenic features (14, 83). In melanoma, the accumulation of highly suppressive Treg subsets, such as TIM-3+ Tregs, CD39+PD-1+ Tregs, and CCR8+ Tregs, is associated with disease progression and poor prognosis (20, 31, 64). The identification of these disease-specific Treg subsets provides opportunities for targeted therapeutic intervention.

Despite these differences, both diseases share a common theme: the Treg compartment is dynamically regulated by the local microenvironment. In both vitiligo and melanoma, the inflammatory or tumor milieu shapes Treg phenotype and function, either driving them toward dysfunction (in vitiligo) or enhancing their suppressive capacity (in melanoma) (14, 21, 59). This environmental influence suggests that therapeutic strategies aimed at modulating the local microenvironment could have profound effects on Treg function and, consequently, on disease outcome.

## 6 Therapeutic opportunities: modulating Tregs in vitiligo and melanoma

The divergent roles of Tregs in vitiligo and melanoma necessitate fundamentally different therapeutic approaches: enhancing Treg function to restore tolerance in vitiligo, and selectively inhibiting or depleting Tregs to unleash anti-tumor immunity in melanoma (10, 17, 18, 22, 44) (**Figure 2**). The development of Treg-based therapies requires a nuanced understanding of Treg biology to achieve disease-specific effects while avoiding systemic immune dysregulation.

**Figure 2:**
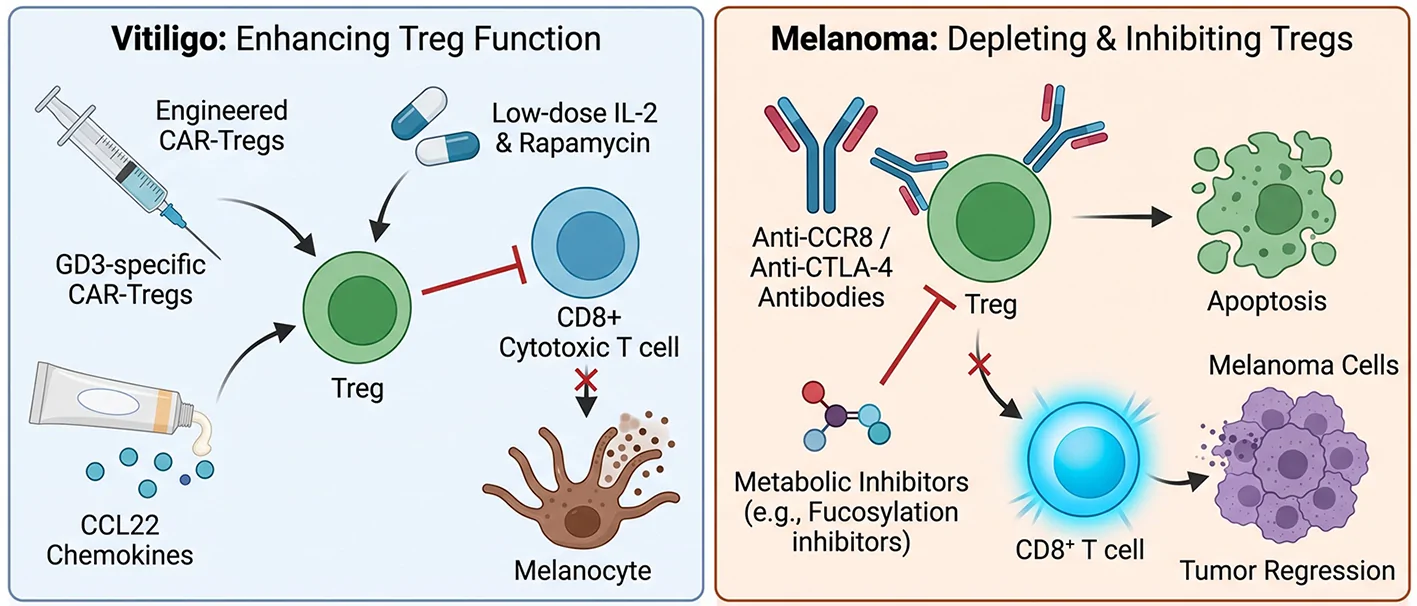
Divergent therapeutic strategies targeting skin-resident regulatory T cells (Tregs) in vitiligo and melanoma. (Left) In vitiligo, therapies aim to enhance Treg numbers and suppressive function to restore immune tolerance. Current and emerging strategies include the adoptive transfer of engineered GD3-specific CAR-Tregs, pharmacological expansion using low-dose IL-2 and rapamycin, and the topical application of CCL22 chemokines to promote local Treg homing to the skin. These approaches strengthen Treg-mediated inhibition of autoreactive CD8+ cytotoxic T cells, thereby preventing the autoimmune destruction of melanocytes. (Right) In melanoma, therapeutic interventions focus on depleting or functionally impairing highly suppressive Tregs to unleash robust anti-tumor immunity. Targeted depletion can be achieved using antibodies against specific surface molecules, such as CCR8 and CTLA-4. Furthermore, metabolic inhibitors, including fucosylation inhibitors, are utilized to compromise Treg proliferation and suppressive stability. By abrogating Treg-mediated immunosuppression, effector CD8+ T cells are effectively unleashed to target and eliminate melanoma cells, ultimately promoting tumor regression.

In vitiligo, therapeutic strategies aimed at replenishing or enhancing Treg function have shown considerable promise. Adoptive transfer of Tregs has been demonstrated to effectively suppress depigmentation in mouse models, with even small numbers of transferred Tregs capable of halting disease progression (36, 56). The development of antigen-specific Tregs represents a particularly attractive approach, as these cells can provide targeted suppression without causing generalized immunosuppression. Ganglioside D3 (GD3)-specific chimeric antigen receptor (CAR) Tregs have been generated and shown to provide superior control of melanocyte-directed cytotoxicity and delay depigmentation compared to non-specific Tregs in a mouse model of spontaneous vitiligo (54). Notably, GD3 is a classic melanoma-associated antigen that is robustly overexpressed in human melanomas. While GD3-specificity is harnessed here to direct CAR-Tregs to suppress local immunity and protect melanocytes in vitiligo, this exact specificity is highly relevant and actively pursued in human oncology, where GD3-directed effector CAR-T cells are being developed to attack melanoma. This shared antigenic target elegantly underscores the mirror-image nature of immunotherapeutic strategies in these two diseases. This approach offers the potential for long-term control of progressive depigmentation through the use of stored, antigen-specific Tregs (54).

Pharmacological approaches to enhance Treg numbers and function in vitiligo include the use of rapamycin, which has been shown to induce lasting remission in mouse models by increasing Treg abundance (56). Low-dose IL-2 therapy is another strategy being explored, as IL-2 is critical for Treg survival and expansion (83). The JAK inhibitor upadacitinib has been reported to downregulate Th1-like Tregs in vitiligo patients, suggesting that modulating the inflammatory environment can restore Treg function (84). Narrow-band UVB phototherapy, a mainstay of vitiligo treatment, has been shown to increase Treg frequency and FOXP3 expression (13). The topical application of CCL22 to promote Treg homing to the skin represents a novel approach that has shown efficacy in mouse models, with treated mice exhibiting significantly reduced depigmentation accompanied by increased Treg infiltration (36, 37). PD-L1 fusion protein therapy has also been shown to reverse depigmentation in vitiligo mice by increasing Treg abundance in the skin (53).

Emerging therapies for vitiligo also include the use of mesenchymal stem cell-derived exosomes. Three-dimensional cultured human umbilical mesenchymal stem cell exosomes (3D-Exos) have been shown to effectively ameliorate vitiligo in mice. They achieve this by delivering specific miRNAs (including *miR-132-3p* and *miR-125b-5p*) to simultaneously potentiate Treg-mediated immunosuppression and suppress oxidative stress-induced melanocyte damage (85). This dual mechanism of action addresses both the immune dysregulation and the oxidative stress components of vitiligo pathogenesis.

In melanoma, therapeutic strategies focus on depleting or functionally impairing Tregs to enhance anti-tumor immunity. Depletion of Tregs using agents such as cyclophosphamide, anti-CD25 antibodies, or anti-CCR4 antibodies has been tested, but clinical efficacy has been limited by the lack of Treg specificity and the risk of inducing autoimmunity (17, 18, 22, 31). Anti-CTLA-4 antibodies, such as ipilimumab, were initially thought to deplete Tregs, but studies in humans have shown that they primarily enhance effector T cell function without significantly depleting intratumoral FOXP3+ cells (76). In contrast, murine studies indicate that Fc-dependent depletion of intratumoral Tregs by macrophages is a key mechanism of anti-CTLA-4 efficacy, suggesting that engineering antibodies with enhanced Fc-mediated effector function could improve Treg depletion in humans (77).

Targeting Treg-specific surface molecules like CCR8 enables selective depletion. Although CCR8 signaling is not required for Treg tumor infiltration, its highly restricted expression on intratumoral Tregs makes it an ideal physical target for antibody-mediated elimination without inducing systemic autoimmunity (31). Targeting the TIGIT/CD226 axis is another approach, as Tregs in melanoma exhibit an increased TIGIT/CD226 ratio, and blocking TIGIT while activating CD226 can disrupt Treg suppression and stability (63).

Metabolic targeting of Tregs is an emerging therapeutic frontier. Inhibitors of fucosylation have been shown to compromise Treg proliferation and suppressive function, leading to reduced tumor growth in mouse models (86). A fucosylation-low (FucoLOW) Treg signature correlates with better prognosis and response to immune checkpoint blockade in melanoma patients (86). Targeting the PRC1.1 complex, which is critical for Treg activation and plasticity, with the selective inhibitor iBP has been shown to enhance anti-tumor immunity and synergize with anti-PD-L1 therapy in melanoma models (87). Similarly, targeting STAT3 to abrogate TIM-3 upregulation on Tregs can improve the response to PD-1 blockade (65).

Combination strategies that incorporate Treg modulation with other immunotherapies are likely to be the most effective. Depleting Tregs in combination with immune checkpoint inhibitors, cancer vaccines, or adoptive T cell therapy has the potential to enhance anti-tumor responses (17, 18, 22). The use of CCL22 to divert Tregs away from tumors and into the skin has been proposed as a strategy to suppress autoimmune side effects of immune checkpoint therapy while limiting Treg accumulation in tumors (38). The identification of Treg-related gene signatures that predict response to immunotherapy may enable patient stratification and personalized treatment approaches (74, 75, 88).

Despite the remarkable promise of these therapeutic modalities, their translational potential is currently hampered by significant clinical challenges and limitations that must be coherently addressed. In the context of vitiligo, while autologous Treg adoptive transfer and engineered CAR-Tregs are conceptually elegant, their broad clinical feasibility is limited by the exorbitant costs, the technical complexity of ex vivo isolation and expansion, and strict regulatory hurdles. Furthermore, a major biological limitation is the risk of lineage instability; transferred Tregs may undergo pathogenic Th1-like conversion upon encountering the hostile, IFN-γ-rich inflammatory microenvironment of the vitiligo skin, thereby exacerbating rather than resolving the disease. Conversely, the paramount challenge in treating melanoma is achieving absolute target selectivity. Historically, generalized systemic Treg depletion (e.g., using anti-CD25 antibodies) has failed clinically due to the rapid induction of severe, life-threatening immune-related adverse events (irAEs) stemming from the breakdown of systemic tolerance. Therefore, the translational success of melanoma interventions strictly hinges on identifying and targeting markers that are exclusively expressed on tumor-infiltrating Tregs (such as CCR8) to decouple anti-tumor efficacy from systemic autoimmune toxicity. Additionally, metabolic interventions face the limitation of off-target effects, as metabolic pathways are often shared between Tregs and crucial anti-tumor effector T cells. Overcoming these specific translational barriers remains the central focus of next-generation immunotherapeutics. A comprehensive head-to-head comparison summarizing the divergent roles, functional alterations, and therapeutic strategies regarding Tregs in vitiligo and melanoma is provided in **Table 2**.

**Table 2:** Summary comparison of skin-resident tregs in vitiligo and melanoma: from pathogenesis to therapeutics.

Feature	Vitiligo (autoimmunity)	Melanoma (tumor immune evasion)
Overall Treg Status	Quantitatively deficient; sparse infiltration in lesional skin.	Overabundant; massive accumulation in the TME.
Predominant Phenotype/Subtypes	Lineage instability; emergence of Th1-like Tregs (T-bet+, IFN-γ+); “exhausted” PD-1+ Tregs.	Hyper-suppressive tumor-infiltrating Tregs (TITRs); CD39+, TIM-3+, TIGIT+, high CTLA-4+.
Chemokine Trafficking	Impaired recruitment due to significantly decreased local CCL22 expression.	Active, aggressive recruitment driven by tumor-derived CCL22 and CCL1.
Primary Functional Defect/Role	Loss of suppressive capacity; failure to halt autoreactive CD8+ T cells and NK cells.	Extreme immunosuppression; acts as a metabolic “IL-2 sink”; potent cytokine secretion (IL-10, TGF-β, IL-35).
Clinical/Prognostic Significance	Decreased Treg frequency correlates with active disease progression and depigmentation severity.	High Treg-to-CD8+ T cell ratio strongly correlates with poor survival. (Note: Development of ICI-induced vitiligo serves as a visible biomarker for robust systemic anti-tumor immunity and prolonged survival).
Therapeutic Goal	Enhance and Replenish: Restore Treg numbers and functional stability to rebuild immune tolerance.	Deplete and Inhibit: Selectively deplete or functionally cripple Tregs to unleash anti-tumor immunity.
Emerging Therapeutic Modalities	Low-dose IL-2, Rapamycin, GD3-specific CAR-Tregs, Topical CCL22, PD-L1 fusion proteins, MSC-derived exosomes (3D-Exos), narrow-band UVB.	Treg-depleting antibodies (e.g., anti-CCR8, Fc-optimized anti-CTLA-4), Metabolic targeting (e.g., fucosylation inhibitors), TIGIT/CD226 modulation.

## 7 Conclusion and future perspectives

The dual role of skin-resident Tregs in vitiligo and melanoma exemplifies the context-dependent nature of immune regulation. In vitiligo, Treg deficiency and dysfunction permit the breakdown of self-tolerance and the development of melanocyte-directed autoimmunity. In melanoma, Treg overabundance and enhanced suppressive function create an immunosuppressive TME that facilitates immune evasion and disease progression. Understanding the mechanistic basis for these opposing outcomes is critical for the rational design of Treg-based therapies.

A critical limitation in current Treg research stems from the discrepancies between preclinical murine models and human clinical data, which presents significant translational challenges. While mouse models are indispensable for mechanistic discoveries, they often fail to perfectly recapitulate the chronic, spontaneous nature of human vitiligo or the complex mutational burden of human melanoma. Anatomically, human skin significantly differs from murine skin in thickness, appendage density, and the composition of resident immune cells. Because skin Tregs preferentially cluster around the hair follicle bulge, the exceptionally high hair follicle density in mice may skew Treg localization and functional dynamics compared to human glabrous or sparsely haired skin (28). Furthermore, contradictory findings frequently arise from species-specific immunological differences. A prime example is anti-CTLA-4 immunotherapy: while murine studies consistently demonstrate that its efficacy heavily relies on Fcγ receptor-mediated depletion of intratumoral Tregs by macrophages (77), corresponding human studies reveal that anti-CTLA-4 primarily drives effector T cell expansion without significantly depleting Tregs (76). Such contradictions underscore the limitation of directly extrapolating murine data to human therapies and highlight the urgent translational need to validate preclinical findings using humanized *in vivo* models and advanced ex vivo human skin explant systems.

Several key questions remain to be addressed. The precise molecular mechanisms that drive Treg instability and Th1-like conversion in vitiligo, as opposed to the maintenance of a highly suppressive state in melanoma, warrant further investigation. The role of the tissue microenvironment in shaping Treg phenotype and function is a central theme that requires deeper exploration, particularly in the context of the skin as a unique immunological niche. The development of more specific markers to distinguish disease-relevant Treg subsets from those involved in maintaining systemic tolerance is essential for advancing therapeutic strategies.

Future research should focus on translating preclinical findings into effective clinical therapies. In vitiligo, clinical trials of Treg-enhancing strategies, including low-dose IL-2, antigen-specific CAR Tregs, and topical chemokine-based therapies, are needed. In melanoma, the development of Treg-depleting antibodies with improved specificity, as well as metabolic inhibitors that selectively target intratumoral Tregs, holds promise for enhancing the efficacy of existing immunotherapies. The integration of Treg modulation with other therapeutic modalities, such as immune checkpoint inhibitors, represents a logical next step.

Ultimately, the divergent roles of Tregs in vitiligo and melanoma underscore the need for disease-specific therapeutic approaches that are informed by a deep understanding of Treg biology in each context. By learning from the mechanistic parallels and divergences between these two diseases, researchers can develop more precise and effective strategies to either enhance or inhibit Treg function, with the goal of restoring immune balance and improving patient outcomes.
